# Exercise-Induced Bronchoconstriction in Children

**DOI:** 10.3389/fmed.2021.814976

**Published:** 2022-01-03

**Authors:** Angela Klain, Cristiana Indolfi, Giulio Dinardo, Marcella Contieri, Fabio Decimo, Michele Miraglia Del Giudice

**Affiliations:** Department of Woman, Child and General and Specialized Surgery, University of Campania “Luigi Vanvitelli”, Naples, Italy

**Keywords:** exercise-induced bronchoconstriction, exercise induced asthma, children, asthma, atopy

## Abstract

Exercise-induced bronchoconstriction (EIB) is a transient airflow obstruction, typically 5–15 min after physical activity. The pathophysiology of EIB is related to the thermal and osmotic changes of the bronchial mucosa, which cause the release of mediators and the development of bronchoconstriction in the airways. EIB in children often causes an important limitation to physical activities and sports. However, by taking appropriate precautions and through adequate pharmacological control of the condition, routine exercise is extremely safe in children. This review aims to raise awareness of EIB by proposing an update, based on the latest studies, on pathological mechanisms, diagnosis, and therapeutic approaches in children.

## Introduction

EIB is a condition of bronchoconstriction of the airways following intense physical activity, that may occur in people with or without bronchial asthma. EIB has been for years defined also with the acronym EIA (exercise-induced asthma). The two terms, however, indicate two different clinical conditions: EIA is a real pathology, characterized by bronchial hyperactivity and chronic inflammation, while the EIB represents the transitory airway narrowing, that may also occur in non-asthmatic patients. The two conditions also differ therapeutically: EIA benefits from corticosteroid treatment to manage the underlying chronic inflammation, while EIB, in most cases, is managed with a short-acting b2-agonist before exercise. Therefore, although the two terms have long been interchangeable, currently, it is preferred to talk about EIB, as most asthmatic people have EIB, but not all patients with EIB have asthma (1).

Symptoms of EIB are not characteristic and include dyspnea, cough, shortness of breath, wheezing, and chest pain. Some clinical conditions such as exercise-induced laryngeal obstruction (EILO), respiratory model disorders, chest wall restrictions, and cardiovascular pathologies, may mimic EIB symptoms. EILO often occurs in young female adolescents with dyspnea with or without stridor during exercise and is detected by laryngoscopy during treadmill test (continuous laryngoscopy exercise) (2). Breathing pattern disorders (the most frequent is the hyperventilation syndrome) are characterized by dyspnea on exertion or at rest, associated or not with organic diseases; the diagnosis requires multidisciplinary evaluation, including a psychophysiological approach (3). Chest wall restrictions are congenital or acquired conditions that limit the expansion of the rib cage, causing a sense of 'air hunger at rest or after physical exertion (4). Cardiovascular pathologies such as supraventricular tachycardia, cardiomyopathy, cyanotic and acyanotic structural congenital heart diseases can cause dyspnea and chest pain (5, 6). Cardiopulmonary exercise test, which measures ventilation, oxygen consumption (VO2), carbon dioxide production (VCO2), pulse oximetry, and flow-volume loops during and after exertion, is essential for identifying forms of dyspnea related to alterations of cardiovascular, pulmonary, and musculoskeletal systems (7).

EIB is observed in 40–90% of asthmatic children, especially in those with severe asthma not pharmacologically controlled (8–11).

Furthermore, EIB is prevalent in athletes who play endurance sports (12), reaching a prevalence of 55% in winter sports athletes (13).

Atopy is the main risk factor, as demonstrated by epidemiological data showing that among children with EIB, up to 40% of them have allergic rhinitis and 30% can develop asthma in the adult, following the march atopic (14).

There is some evidence that supports that eosinophilic airway inflammation is associated with EIB in asthmatic children and FeNO (Fractional Exhaled Nitric Oxide) levels correlate to EIB severity (15). According to some authors, FeNO values could be used as a predictor of EIB in asthmatic children (16), in particular, low eNO may be used as a negative predictor for EIB (17). According to Buchvald et al. EIB could be excluded with a probability of 90% for FeNO50 levels <20 p.p.b., in subjects not currently using inhaled corticosteroids, and <12 p.p.b. in those currently using inhaled corticosteroids (18).

In the pediatric age, atopic dermatitis (11), sensitization to indoor allergens (19), elevated levels of both seasonal and perennial IgE (20) appear to be associated with an increased risk of developing EIB.

Environmental factors such as exposure to cold air, high atmospheric pressures, humidity, and pollutants are related to an increased risk of developing EIB (21–23).

## Pathophysiology and Mechanisms Underlying EIB

When triggering factors are present, such as cold and dry air, pollutants, allergens, exercise causes increased ventilation causing dehydration of the airways mucosa, resulting in increased osmolarity, contraction of bronchial smooth muscle, and an influx of eosinophils and mast cells that release inflammatory mediators (leukotrienes, histamine, IL-8, tryptase, and prostaglandins) (24). These signaling molecules increase airway smooth muscle contraction, mucus production, microvascular permeability, and sensory nerve activation, representing the primary stimulus for bronchoconstriction and airway edema (25, 26).

**Figure 1:**
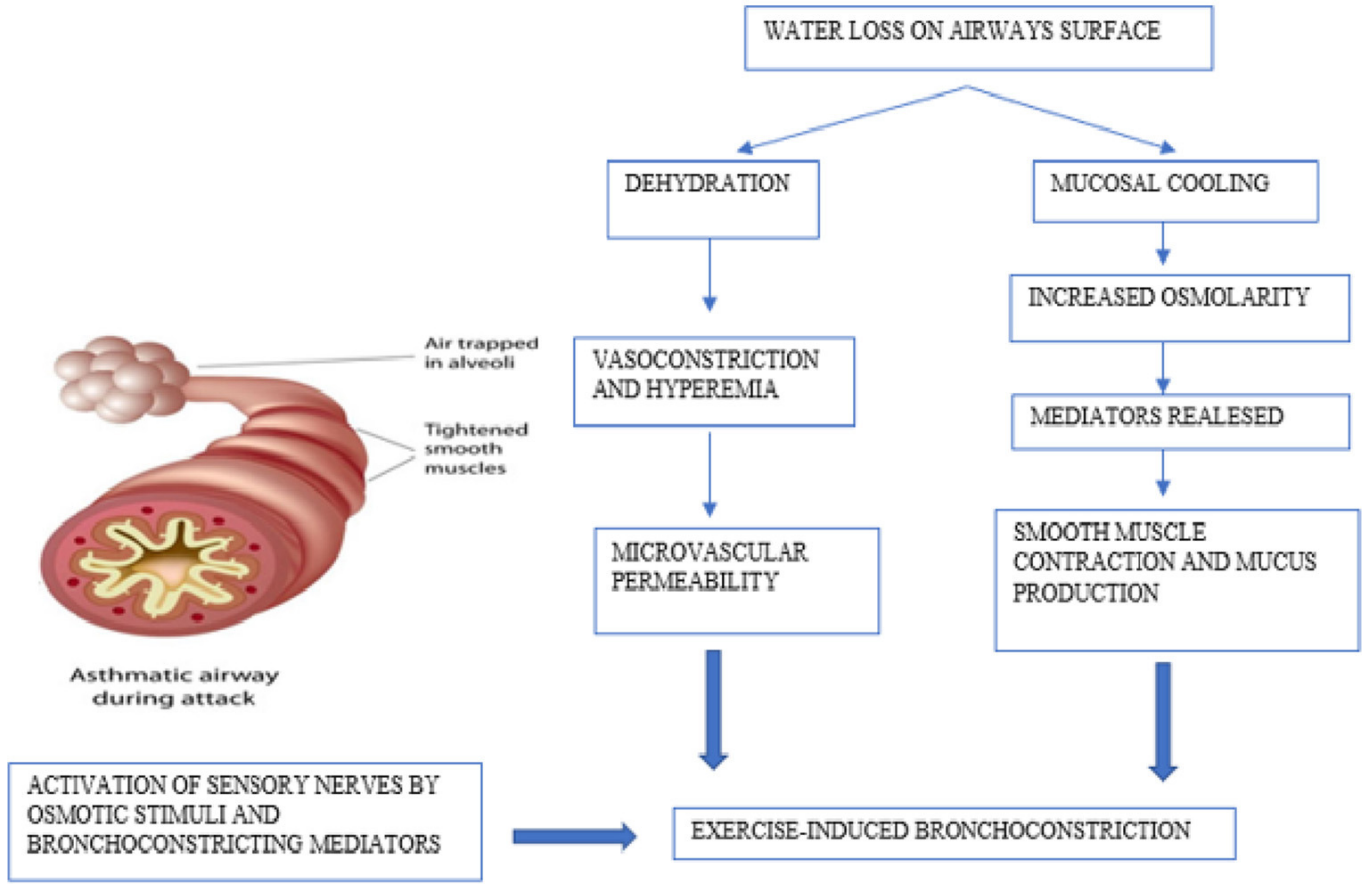
Pathophysiology and mechanisms underlying exercise-induced bronchoconstriction.

Neurological factors are also implicated in the pathophysiology of EIB. The sensory nerves are activated directly by osmotic stimuli; in addition, prostaglandins can also directly activate or alter the activation threshold of sensory nerves. In the UK, Maher et al. have shown that PGD2, which is predominantly produced by mast cells, initiates the activation of sensory nerves *via* the DP1 receptor, regulating the tone of the airways (27). Furthermore, there is evidence that neurokinin A and Cys-LT levels in the airways are correlated with the degree of bronchoconstriction after exercise and with the release of mucins, particularly MUC5AC which is more expressed in the sputum of patients with bronchoconstriction after exercise (28) (Figure 1).

## Diagnosis

In 2019 Lammers et al. conducted a study about the ability of pediatricians to assess EIB from anamnesis and physical examination: the sensitivity of a pediatrician's predicted diagnosis of BEI was 84%, compared with a specificity of 24%; EIB severity prediction was poor, with many serious cases underestimated (29). In 2020 the same authors have studied the ability of pediatricians to assess EIB from post-exercise videos, comparing it to EIB severity assessed by the exercise challenge test (ECT) (30). The study found a positive predictive value for pediatricians' diagnosis of BEI of 61% and a negative predictive value was 60%. These studies show that pediatricians have at best a fair ability to assess EIB severity from symptoms and post-exercise videos, implicating that standardized ECT is still fundamental in the diagnosis of EIB.

Spirometry after exercise challenge test is considered the gold standard for the diagnosis of EIB. According to ATS (American Thoracic Society)/European Respiratory Society guidelines, at least two FEV1 (forced expiratory volume in the 1st s) maneuvers must be measured pre and after exercise challenge (e.g., treadmill). FEV1 is usually measured at 5, 10, 15, and 30 min after exercise. A difference between the pre-exercise FEV1 value and the lowest FEV1 value recorded within 30 min after exercise >10% is diagnostic of EIB (24, 31, 32).

There are alternative tests to ECT to be used in situations where, for example, the patient cannot perform exercise tests: methacholine challenge, eucapnic voluntary hyperventilation, mannitol challenge, hypertonic saline challenge, histamine challenge (33). Nowadays, the role of these tests is still debated.

Recently, a Dutch study investigated the role of surface electromyography (EMG) to study the electrical activity of the diaphragm, as an alternative EIB diagnostic tool: EMG peak amplitudes measured at the diaphragm increased significantly more in children with EIB compared to children without EIB, suggesting a role for EMG as a non-invasive and stress-independent test to be practiced in cases where ECT is not feasible (34).

At present, there is no sufficient evidence to support the widespread adoption of any existing EIB screening tools (35).

## Treatment

In patients with EIB, ATS recommends the use of a short-acting b2-agonist (SABA) 5–20 min before exercise. For patients who continue to have symptoms of EIB despite the administration of SABA before exercise, daily inhaled corticosteroid, daily leukotriene receptor antagonist, or mast cell stabilizing agent before exercise should be used. In allergic patients with BEI, ATS recommends the use of antihistamines in addition to therapy with SABA (24).

EIB in asthmatic children often causes an important limitation to physical activities and sports. However, by taking appropriate precautions (avoiding or reducing activity in highly polluted areas, exposure to cold air, performing a pre-exercise warm-up and regular physical activity, and through adequate pharmacological control of asthma), exercise routinely is extremely safe in children (36). Some studies have shown that regular continuous aerobic exercise benefits asthmatic patients on FEV1, PEF, FVC, FEF 25–75% and, in general, improves patients' symptoms and quality of life (37). In childhood, EIA causes QoL (quality of life) impairment (38). A Swedish study of adolescents with or without asthma demonstrated a significant association between spirometry-defined EIB and reduced QoL (39). Routine physical exercise improves childhood EIB (including QoL) and, therefore, should be recommended as a supplementary therapy to drugs (36).

**Figure 2:**
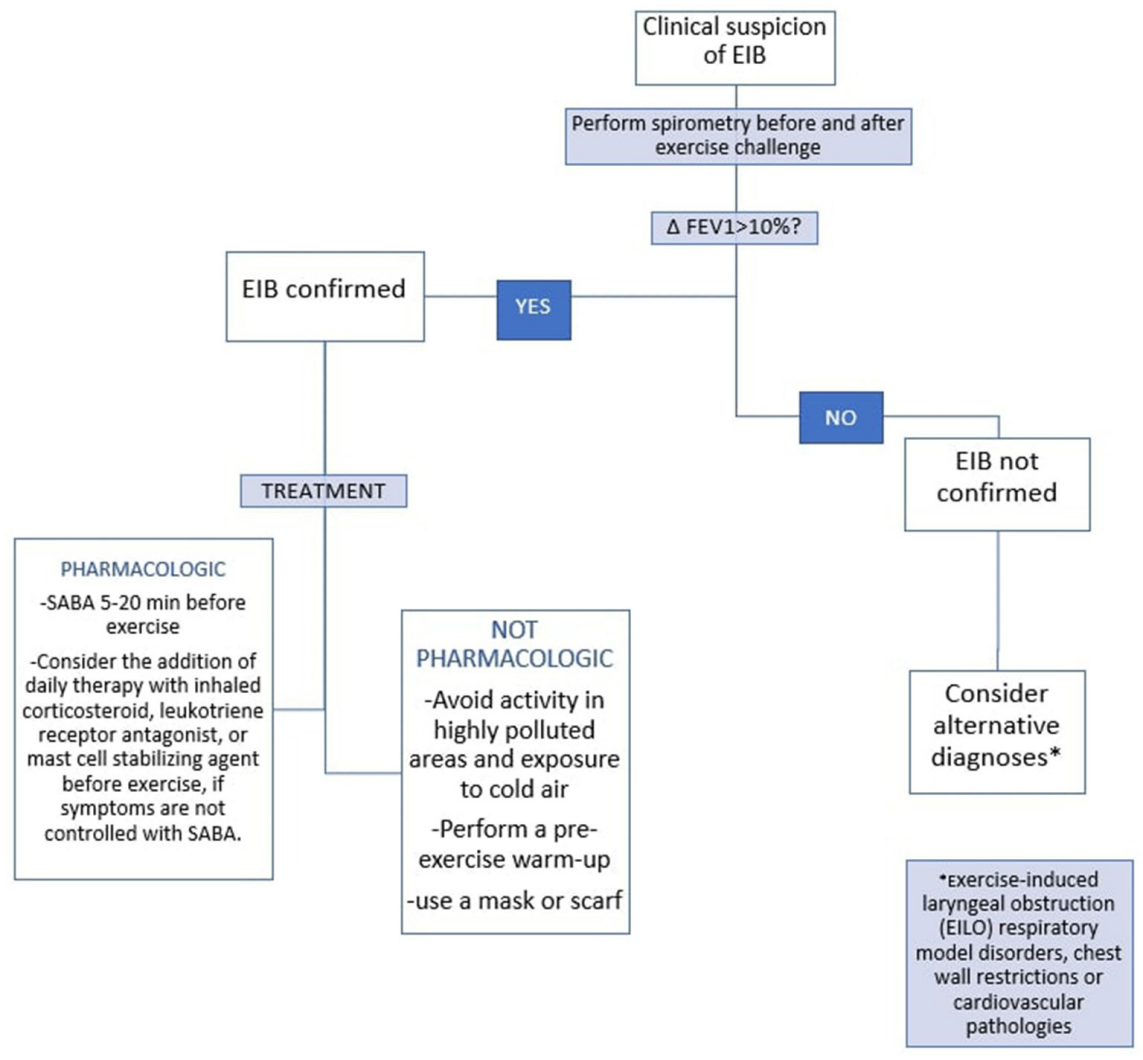
EIB flow chart in children, according to ATS.

In Figure 2 the diagnostic and therapeutic pathway of EIB in children is summarized according to the recommendations of the ATS (Figure 2).

## Conclusion

EIB is a very common condition in children and is related to the main atopic risk factors. In most cases, the symptoms are mild and are controlled through SABA therapy before exercise and regular physical activity. It is important to remember that untreated or mistreated EIB can have an impact on physical and psychological child growth and, therefore, requires pediatricians well-acquainted with an early recognition and therapy education of children and their families.

## Author Contributions

AK, CI, GD, MC, FD, and MM participated equally in the drafting of the manuscript. All authors contributed to the article and approved the submitted version.

## Conflict of Interest

The authors declare that the research was conducted in the absence of any commercial or financial relationships that could be construed as a potential conflict of interest.

## Publisher's Note

All claims expressed in this article are solely those of the authors and do not necessarily represent those of their affiliated organizations, or those of the publisher, the editors and the reviewers. Any product that may be evaluated in this article, or claim that may be made by its manufacturer, is not guaranteed or endorsed by the publisher.
